# Plasma free amino acid profiles evaluate risk of metabolic syndrome, diabetes, dyslipidemia, and hypertension in a large Asian population

**DOI:** 10.1186/s12199-017-0642-7

**Published:** 2017-04-07

**Authors:** Natsu Yamaguchi, MH Mahbub, Hidekazu Takahashi, Ryosuke Hase, Yasutaka Ishimaru, Hiroshi Sunagawa, Hiroki Amano, Mikiko Kobayashi- Miura, Hideyuki Kanda, Yasuyuki Fujita, Hiroshi Yamamoto, Mai Yamamoto, Shinya Kikuchi, Atsuko Ikeda, Mariko Takasu, Naoko Kageyama, Mina Nakamura, Tsuyoshi Tanabe

**Affiliations:** 1 Department of Public Health and Preventive Medicine, Graduate School of Medicine, Yamaguchi University, 1-1-1 Minami-Kogushi, Ube, Yamaguchi 755-8505 Japan; 2 Division of Health Administration and Promotion, Graduate School of Medicine, Tottori University, Yonago, Japan; 3 Department of Biochemistry, Shimane University Faculty of Medicine, Izumo, Japan; 4 Department of Environmental Medicine and Public Health, Faculty of Medicine, Shimane University, Izumo, Japan; 5 Institute for Innovation, Ajinomoto Co., Inc., Kawasaki, Japan

**Keywords:** Amino acids, Lifestyle-related diseases, Metabolic syndrome, Diabetes mellitus, Dyslipidemia, Hypertension

## Abstract

**Background:**

Recently, the association of plasma free amino acid (PFAA) profile and lifestyle-related diseases has been reported. However, few studies have been reported in large Asian populations, about the usefulness of PFAAs for evaluating disease risks. We examined the ability of PFAA profiles to evaluate lifestyle-related diseases in so far the largest Asian population.

**Methods:**

We examined plasma concentrations of 19 amino acids in 8589 Japanese subjects, and determined the association with variables associated with obesity, blood glucose, lipid, and blood pressure. We also evaluated the PFAA indexes that reflect visceral fat obesity and insulin resistance. The contribution of single PFAA level and relevant PFAA indexes was also examined in the risk assessment of lifestyle-related diseases.

**Results:**

Of the 19 amino acids, branched-chain amino acids and aromatic amino acids showed association with obesity and lipid variables. The PFAA index related to visceral fat obesity showed relatively higher correlation with variables than that of any PFAA. In the evaluation of lifestyle-related disease risks, the odds ratios of the PFAA index related to visceral fat obesity or insulin resistance with the diseases were higher than most of those of individual amino acid levels even after adjusting for potential confounding factors. The association pattern of the indexes and PFAA with each lifestyle-related disease was distinct.

**Conclusions:**

We confirmed the usefulness of PFAA profiles and indexes as markers for evaluating the risks of lifestyle-related diseases, including diabetes mellitus, metabolic syndrome, dyslipidemia, and hypertension in a large Asian population.

## Background

Lifestyle-related diseases are pathophysiological states that include metabolic syndrome, diabetes mellitus (DM), dyslipidemia, hypertension and gout, and often lead to cardiovascular disease [1, 2]. Excess visceral fat and hyperinsulinemia are regarded as risk factors of lifestyle-related diseases [3, 4], and early detection of such factors is important for prevention of the diseases. Because of a shift towards the Western lifestyle and diet, the prevalence of lifestyle-related diseases has significantly increased in Asian populations [2]. For early detection of lifestyle-related diseases, the development of useful biomarkers is essential which would also help in better understanding of the disease pathophysiology and early intervention to avoid the progression of diseases and deterioration of patients’ conditions [5].

Recent reports have shown that plasma free amino acid (PFAA) profile can serve as an effective biomarker for detection of lifestyle-related diseases [6–9]. PFAA profile was also reported to be useful for identifying diabetic patients at risk of developing cardiovascular diseases [10, 11]. Although these reports suggested that changes of amino acid metabolism play important roles in the pathogenesis of lifestyle-related diseases, the number of subjects in those studies was limited. Furthermore, we previously reported the construction of PFAA index 1 and index 2, which evaluated visceral fat area (VFA) and insulin resistance, respectively, and revealed their association with variables of lifestyle-related diseases and usefulness of such indexes for predicting the future risk of developing the diseases including metabolic syndrome, DM, dyslipidemia, and hypertension [12]. However, it is necessary to confirm the capabilities of the PFAA indexes to evaluate the risk of lifestyle-related diseases in a large Japanese population.

Therefore, the purpose of this study was to examine the ability of PFAA and relevant indexes for evaluating lifestyle-related diseases in so far the largest sample of an Asian population for such type of studies.

## Methods

### Subjects

The study was conducted in accordance with the Declaration of Helsinki, and the institutional review board of Shimane University (20100129-3) and Yamaguchi University (H25-26-2) approved the current study protocol. A total of 8589 Japanese subjects who underwent the health examination during 2009 to 2011 in Shimane prefecture and gave informed consent to participate in the study, were included.

### Measurement of metabolic variables and quantification of PFAAs

Blood samples were taken from the subjects after an 8-h fast. Serum levels of total cholesterol (T-CHO), high-density lipoprotein cholesterol (HDL-C), low- density lipoprotein cholesterol (LDL-C), and triglyceride (TG) were determined enzymatically. Free plasma glucose (FPG) was measured with the hexokinase method, and HbA1c was determined using the latex agglutination immunoassay. Plasma amino acid concentrations were analyzed following the protocol previously described elsewhere [12–16]. Briefly, blood samples (5 mL) were collected from forearm veins after overnight fasting into tubes containing disodium ethylenediaminetetraacetate that were immediately placed on ice. The plasma was prepared by centrifugation at 3000 r.p.m. at 4 °C for 15 min and then stored at −80 °C until analysis. The plasma amino acid concentrations were measured by high-performance liquid chromatography–electrospray ionization mass spectrometry followed by precolumn derivatization as previously described [13–16]. The following 19 amino acids were measured: Alanine (Ala), Arginine (Arg), Asparagine (Asn), Citrulline (Cit), Glutamine (Gln), Glycine (Gly), Histidine (His), Isoleucine (Ile), Leucine (Leu), Lysine (Lys), Methionine (Met), Ornithine (Orn), Phenylalanine (Phe), Proline (Pro), Serine (Ser), Threonine (Thr), Tryptophane (Trp), Tyrosine (Tyr), and Valine (Val). We did not perform the measurements of other genetically-encoded amino acids like glutamate, aspartate, and cysteine due to their instability in the blood [14].

### Clinical assessment

In this study, metabolic syndrome was defined according to the following Japanese diagnostic criteria for the syndrome: visceral obesity (waist ≥ 85 cm in males and ≥90 cm in females) plus at least 2 of the following three components: (1) HDL-C < 40 mg/dL, TG ≥ 150 mg/dL, or the use of medication for dyslipidemia; (2) FPG ≥ 110 mg/dL or the use of medication for DM; and (3) blood pressure ≥130/85 mmHg or the use of antihypertensive medication. DM was defined in patients with FPG ≥126 mg/dL, HbA1c ≥ 6.5%, or those who were taking medication for DM. Dyslipidemia was defined in individuals with fasting LDL-C ≥ 140 mg/dL, HDL-C < 40 mg/dL, TG ≥ 150 mg/dL, or those who were taking medication for dyslipidemia. Hypertension was defined in patients with systolic blood pressure (SBP) ≥140 or diastolic blood pressure (DBP) ≥ 90 mmHg or those who were taking antihypertensive medications.

### Calculation of PFAA indexes

In this study, we used the amino acid index 1 and index 2 constructed and validated in a previous study [12]. The amino acid index 1 is the multiple linear regression model with variable selection to model the relationships between the PFAA profiles with the visceral fat area, consisting of Leu, Ala, Tyr, Asn, Trp, and Gly. The amino acid index 2 is the multiple linear regression model with variable selection to model the relationships between the PFAA profiles with 2-h post-challenge insulin levels (Ins120 min), consisting of Ile, Ala, Tyr, Phe, Met, and His. Therefore, each of these PFAA indexes (index 1 and index 2) is a single dimension that contains information on multidimensional PFAA profiles. Such compression of information on PFAA profiles allows maximization of the discrimination between patients and control subjects.

### Correlation between PFAAs and metabolic variables

Correlation analysis between each of single PFAA concentration and PFAA indexes and metabolic variables was performed as previously described [12] by using the Pearson product-moment correlation coefficient. In addition, two-dimensional hierarchical cluster analysis that was based on the correlation coefficient matrix between the PFAA concentrations and additional measured variables was performed. For each single PFAA and the PFAA indexes, three different models were used with or without adjustments for variables as follows: model 1) without adjusting, model 2) adjusted for age and gender, model 3) adjusted for age, gender, and body mass index (BMI).

### Association between PFAAs and lifestyle related diseases

We examined the relationships of the PFAA profiles to lifestyle-related diseases to determine whether each PFAA index and single PFAA concentration were related to DM, metabolic syndrome, dyslipidemia, and hypertension. The PFAA indexes and all of the amino acids were scaled to multiples of 1 SD. A logistic regression analysis was used to assess the contribution of each PFAA index and single PFAA concentration as continuous variables in the evaluation of these diseases. The logistic regression analysis was performed with adjustment for age and gender. Furthermore, to exclude the cross-over effects among diseases on the single PFAA level and each PFAA index, further adjustments were performed as follows: metabolic syndrome for age, gender and BMI; DM for age, gender, BMI, LDL-C, HDL-C, TG, SBP and DBP; dyslipidemia for age, gender, BMI, FPG, HbA1c, SBP and DBP; hypertension for age, gender, BMI, FPG, HbA1c, LDL-C, HDL-C and TG.

A two-sided probability value of *p* < 0.01 was considered to be statistically significant. R version 3.1.3 [R Core Team (2015). R: A language and environment for statistical computing. R Foundation for Statistical Computing, Vienna, Austria] was used for the statistical analyses. All of the data were analyzed anonymously throughout the study.

## Results

The numbers of subjects and their demographic and clinical characteristics have been presented in Table 1. Significant differences (*p* < 0.001 to 0.01) between the diseased and non-diseased populations were observed for all variables except for LDL cholesterol for DM and hypertension.

**Table 1:** Demographic and clinical characteristics of the study subjects. Values are expressed as means ± standard deviations for continuous variables

	Metabolic syndrome		DM		Dyslipidemia		Hypertension	
Characteristics	Disease	Non-disease	Disease	Non-disease	Disease	Non-disease	Disease	Non-disease
N, subjects	737	6433	465	7650	3582	4533	3141	4974
(male, female)	(539, 198)	(2915, 3518)	(284, 181)	(3555, 4095)	(1708, 1874)	(2131, 2402)	(1613, 1528)	(2226, 2748)
Age	58.6 ± 11.7	52.7 ± 15.3***	65.4 ± 11.9	55.9 ± 16.8***	60.2 ± 14.4	53.5 ± 17.7***	65.1 ± 13.0	51.0 ± 16.4***
BMI (kg/m^2^)	26.9 ± 3.1	22.3 ± 3.1***	24.2 ± 3.8	22.7 ± 3.3***	23.6 ± 3.3	22.1 ± 3.3***	23.7 ± 3.5	22.1 ± 3.2***
Waist (cm)	93.5 ± 6.6	79.7 ± 8.6***	86.7 ± 9.5	80.8 ± 9.3***	84.3 ± 8.7	78.7 ± 9.2***	85.0 ± 9.1	79.1 ± 8.9***
FPG (mg/dL)	114 ± 34	95 ± 13***	136 ± 45	94 ± 10***	99 ± 20	95 ± 15***	101 ± 21	94 ± 15***
HbA1c (%)	6.3 ± 1.2	5.6 ± 0.5***	7.3 ± 1.3	5.6 ± 0.4***	5.9 ± 0.8	5.6 ± 0.5***	5.9 ± 0.8	5.6 ± 0.6***
TG (mg/dL)	170 ± 102	93 ± 61***	128 ± 101	98 ± 65***	133 ± 86	74 ± 28***	113 ± 76	92 ± 60***
HDL-C (mg/dL)	54 ± 15	66 ± 17***	58 ± 16	65 ± 17***	59 ± 16	69 ± 16***	62 ± 16	66 ± 17***
LDL-C (mg/dL)	125 ± 32	121 ± 31***	119 ± 32	120 ± 30	137 ± 33	107 ± 20***	121 ± 30	120 ± 31
SBP (mmHg)	140 ± 15	125 ± 16***	135 ± 18	127 ± 17***	131 ± 17	124 ± 16***	141 ± 16	119 ± 11***
DBP (mmHg)	87 ± 10	77 ± 11***	79 ± 12	78 ± 11**	79 ± 11	76 ± 11***	84 ± 12	73 ± 9***

*BMI* body mass index, *FPG* Free plasma glucose, *HbA1c* haemoglobin A1c, *TG* triglyceride, *HDL-C* high-density lipoprotein cholesterol, *LDL-C* low-density lipoprotein cholesterol, *SBP* systolic blood pressure, *DBP* diastolic blood pressure

Differences in the total number of total subjects for different diseases are due to varying missing data

Statistical differences between disease and non-disease subjects are shown as **p < 0.01, and ***p < 0.001, respectively

Amino acid concentrations in the study populations are shown in Table 2. Concentrations of PFAA differed significantly between the diseased and non-diseased subjects for metabolic syndrome, DM, dyslipidemia, and hypertension (*p* < 0.001 to 0.05). Overall, the concentrations of PFAA were higher in the diseased compared to the non-diseased; however, the concentrations of Gly and Ser for all diseases (except Ser for DM) and Asn for dyslipidemia showed the opposite trend.

**Table 2:** Plasma free amino acid concentrations (μmol/L) and index values in the study population. The values are shown as means ± standard deviations

	Metabolic syndrome		DM		Dyslipidemia		Hypertension	
	Disease	Non-disease	Disease	Non-disease	Disease	Non-disease	Disease	Non-disease
N, subjects	737	6433	465	7650	3582	4533	3141	4974
Val	238.4 ± 38.6	202.8 ± 38.3***	232.3 ± 42.1	204.5 ± 38.9***	215.7 ± 40.0	198.5 ± 37.6***	212.8 ± 39.2	201.9 ± 39.3***
Leu	129.1 ± 24.1	109.5 ± 22.2***	124.0 ± 24.7	110.1 ± 22.6***	115.9 ± 23.7	106.9 ± 21.6***	113.5 ± 22.7	109.2 ± 23.0***
Ile	67.3 ± 15.0	55.6 ± 13.0***	65.1 ± 15.2	56.3 ± 13.4***	59.4 ± 14.3	54.7 ± 12.7***	58.7 ± 13.6	55.5 ± 13.6***
Trp	57.3 ± 10.0	53.0 ± 9.4***	53.9 ± 10.5	53.1 ± 9.4	54.3 ± 9.6	52.2 ± 9.3***	53.7 ± 9.8	52.7 ± 9.3***
Phe	62.0 ± 9.1	56.5 ± 8.9***	61.7 ± 9.0	57.3 ± 9.1***	58.8 ± 8.9	56.6 ± 9.3***	59.9 ± 9.4	56.1 ± 8.8***
Tyr	71.1 ± 13.2	61.3 ± 12.3***	68.6 ± 15.1	62.5 ± 12.7***	64.7 ± 12.6	61.5 ± 13.0***	66.6 ± 13.4	60.5 ± 12.0***
Ala	399.3 ± 78.7	329.9 ± 75.6***	381.5 ± 84.9	334.8 ± 76.8***	352.9 ± 80.4	325.2 ± 73.9***	350.7 ± 79.3	329.1 ± 76.1***
Gly	199.0 ± 46.3	225.6 ± 59.6***	205.1 ± 50.5	224.4 ± 60.0***	219.2 ± 60.2	226.6 ± 59.0***	217.6 ± 60.5	226.9 ± 58.9***
Gln	587.1 ± 76.0	589.1 ± 67.7	598.4 ± 82.7	592.2 ± 68.8	597.2 ± 68.5	588.9 ± 70.5***	597.4 ± 71.2	589.5 ± 68.6***
Ser	106.1 ± 19.9	113.2 ± 20.5***	113.0 ± 21.6	112.3 ± 20.4	110.0 ± 20.2	114.1 ± 20.4***	109.3 ± 20.0	114.2 ± 20.5***
His	82.4 ± 17.8	79.3 ± 14.5***	80.4 ± 9.8	79.2 ± 14.6	79.8 ± 11.8	78.8 ± 16.1**	79.0 ± 9.5	79.3 ± 16.7
Lys	199.4 ± 31.5	181.8 ± 32.6***	195.9 ± 32.6	182.6 ± 32.5***	189.3 ± 31.9	178.6 ± 32.5***	186.9 ± 31.5	181.1 ± 33.2***
Met	27.1 ± 5.1	24.7 ± 5.7***	26.5 ± 5.3	24.8 ± 5.5***	25.1 ± 5.8	24.8 ± 5.3**	25.3 ± 4.9	24.7 ± 5.9***
Thr	130.0 ± 31.9	122.0 ± 27.1***	125.2 ± 29.3	121.9 ± 27.3*	122.1 ± 27.5	122.2 ± 27.4	123.3 ± 28.4	121.4 ± 26.8**
Asn	45.6 ± 7.0	45.7 ± 7.2	46.7 ± 8.1	45.6 ± 7.2**	45.3 ± 6.8	46.0 ± 7.5***	45.6 ± 7.3	45.7 ± 7.2
Cit	30.6 ± 8.2	30.0 ± 7.4*	34.0 ± 11.4	30.7 ± 7.9***	31.7 ± 8.0	30.3 ± 8.3***	32.8 ± 8.6	29.7 ± 7.6***
Arg	98.4 ± 17.9	94.5 ± 18.8***	100.1 ± 22.1	95.0 ± 18.5***	97.1 ± 18.2	93.9 ± 19.1***	97.2 ± 17.7	94.1 ± 19.3***
Pro	153.9 ± 45.7	128.0 ± 42.2***	148.1 ± 46.5	129.4 ± 42.7***	135.5 ± 44.5	126.5 ± 41.7***	134.1 ± 42.1	128.2 ± 43.7***
Orn	51.7 ± 11.6	47.1 ± 11.9***	54.8 ± 15.3	48.2 ± 12.6***	50.6 ± 12.6	46.9 ± 12.7***	51.6 ± 12.9	46.6 ± 12.4***
Index 1	143.3 ± 35.1	101.0 ± 34.8***	133.3 ± 39.9	104.3 ± 36.0***	115.3 ± 37.7	98.6 ± 34.5***	116.1 ± 37.4	99.6 ± 35.0***
Index 2	50.5 ± 17.8	34.5 ± 17.0***	48.3 ± 17.6	36.5 ± 17.6***	41.3 ± 17.2	34.0 ± 17.6***	42.9 ± 16.3	33.6 ± 17.8***

Differences in the total number of total subjects for different diseases are due to varying missing data

Statistical differences between disease and non-disease subjects are shown as *p < 0.05, **p < 0.01, and ***p < 0.001, respectively

We first examined the relationship between variables that were associated with lifestyle-related diseases and amino acids measured in this study (Table 3). The variables are obesity-related (BMI, waist circumference), glucose-related (FPG, HbA1c), lipid-related (HDL-C, LDL-C, and TG), and blood pressure-related (SBP, DBP). Branched-chain amino acids (BCAA; Val, Leu, Ile) showed moderately positive correlation with both obesity variables and with TG. All other correlations of BCAA with the rest variables were positive but weak. Like BCAA, aromatic amino acids (AAA; Tyr, Phe, Trp) also showed similar trends of correlations: positive (moderate to weak) correlation with both obesity variables and with TG, and weakly positive correlations with others. Among gluconeogenic amino acids (Ala, Gly, Gln, Ser), Ala showed moderately positive correlation with several variables including BMI, waist circumstance, blood glucose, and TG. Conversely, the correlation of Gly, Gln, and Ser was very weak or negative with almost all variables. Correlations of other single amino acids with lifestyle-related diseases were either very low or did not show any specific trend. Both PFAA indexes exhibited moderate to weak positive correlations with most of the variables associated with lifestyle-related diseases. Overall, the correlations were higher for the PFAA indexes compared with those for the single amino acids.

**Table 3:** Correlation between PFAA profiles and metabolic variables

		Obesity		Glucose		Lipid		Blood pressure	
	Age	BMI	Waist circumstance	FPG	HbA1c	LDL	TG	SBP	DBP
Val	0.008	0.391	0.406	0.247	0.199	0.142	0.341	0.151	0.190
Leu	−0.052	0.371	0.377	0.223	0.162	0.117	0.340	0.109	0.198
Ile	−0.035	0.327	0.336	0.219	0.162	0.056	0.353	0.123	0.184
Trp	−0.064	0.219	0.230	0.109	0.033	0.045	0.254	0.076	0.173
Phe	0.278	0.255	0.309	0.177	0.200	0.028	0.156	0.176	0.140
Tyr	0.282	0.325	0.393	0.212	0.183	0.028	0.201	0.233	0.195
Ala	−0.030	0.331	0.337	0.300	0.171	0.089	0.328	0.183	0.226
Gly	0.035	−0.174	−0.183	−0.108	−0.088	−0.039	−0.163	−0.084	−0.125
Gln	0.243	0.033	0.075	0.001	0.072	0.054	−0.075	0.044	−0.019
Ser	−0.051	−0.136	−0.175	−0.047	−0.040	−0.061	−0.264	−0.144	−0.156
His	−0.062	0.121	0.118	0.063	0.006	0.027	0.118	0.010	0.079
Lys	0.108	0.219	0.262	0.181	0.157	0.123	0.158	0.106	0.140
Met	−0.012	0.166	0.172	0.151	0.046	−0.059	0.116	0.069	0.133
Thr	−0.080	0.102	0.088	0.087	−0.015	−0.125	0.071	0.052	0.114
Asn	0.008	−0.014	−0.010	0.064	0.000	−0.091	−0.017	0.000	0.035
Cit	0.460	−0.104	−0.017	0.060	0.176	−0.034	0.018	0.139	−0.009
Arg	0.141	0.047	0.094	0.146	0.122	0.063	0.047	0.091	0.093
Pro	−0.073	0.200	0.204	0.160	0.080	−0.002	0.235	0.078	0.129
Orn	0.376	0.091	0.171	0.151	0.200	0.019	0.104	0.166	0.057
Index 1	0.093	0.463	0.494	0.310	0.252	0.135	0.379	0.242	0.261
Index 2	0.255	0.352	0.403	0.249	0.259	0.097	0.291	0.247	0.187
N, subjects	8588	8589	7170	7324	8004	8023	8023	8115	8115

Pearson’s correlation coefficients were calculated, and hierarchical clustering was conducted

*BMI* Body mass index, *FPG* Fasting plasma glucose, *HbA1c* Hemoglobin A1c, *LDL-CHO* LDL cholesterol, *TG* Triglyceride, *SBP* Systolic blood pressure, *DBP* Diastolic blood pressure

index 1 and index 2: the Indexes generated by plasma free amino acid profiles

We then examined the capability of PFAA to evaluate the risk of metabolic syndrome, DM, dyslipidemia, and hypertension after adjustments for age and gender (Table 4). All the BCAA showed significant positive associations with all four diseases examined [OR between 1.23 and 2.16; 95% CI between 1.15 and 1.98 (lower) and 1.30 to 2.36 (upper); *p* < 0.001]. Especially, the odds ratios of BCAA with metabolic syndrome, DM and dyslipidemia were high, at least 1.7. Also, the odds ratios of AAA showed significant positive association with all four diseases [OR between 1.11 and 1.65; 95% CI between 1.05 and 1.52 (lower) and 1.17 to 1.78 (upper); *p* < 0.001] except Trp in DM. The odds ratios shown by AAA were relatively low compared with those by BCAA. Gluconeogenesis-related amino acids showed a typical pattern of odds ratio for all the four diseases, positive association for Ala [OR between 1.37 and 2.15; 95% CI between 1.30 and 1.52 (lower) and 1.98 to 2.33 (upper); *p* < 0.001] and inverse association for Gly, Gln, and Ser [OR between 0.57 and 0.85; 95% CI between 0.51 and 0.80 (lower) and 0.64 to 0.89 (upper); *p* < 0.001] except for Ser in DM and Gln in dyslipidemia (Table 3). Then we confirmed the capabilities of the PFAA index 1 [OR between 1.52 and 3.23; 95% CI between 1.44 and 2.92 (lower) and 1.61 to 3.57 (upper); *p* < 0.001] and index 2 [OR between 1.48 and 2.37; 95% CI between 1.39 and 2.16 (lower) and 1.57 to 2.61 (upper); *p* < 0.001] to evaluate the significant positive association with lifestyle-related diseases (Table 4). The index 1 showed higher odds ratio than that of any amino acid with metabolic syndrome, DM, and hypertension. The index 2 showed higher odds ratio than that of each amino acid with metabolic syndrome and hypertension.

**Table 4:** The odds ratios for evaluating metabolic syndrome, DM, dyslipidemia, or hypertension adjusting for age and gender

	Metabolic syndrome			DM			Dyslipidemia			Hypertension		
	Odds ratio		*p*-value	Odds ratio		*p*-value	Odds ratio		*p*-value	Odds ratio		*p*-value
Val	2.16	(1.98–2.36)	<0.001	1.91	(1.73–2.11)	<0.001	1.79	(1.69–1.89)	<0.001	1.30	(1.23–1.37)	<0.001
Leu	2.04	(1.86–2.23)	<0.001	1.82	(1.63–2.02)	<0.001	1.88	(1.77–2.00)	<0.001	1.23	(1.15–1.30)	<0.001
Ile	1.97	(1.80–2.15)	<0.001	1.75	(1.58–1.93)	<0.001	1.69	(1.59–1.79)	<0.001	1.27	(1.20–1.35)	<0.001
Trp	1.27	(1.17–1.37)	<0.001	0.99	(0.89–1.10)	0.859	1.36	(1.29–1.43)	<0.001	1.11	(1.05–1.17)	<0.001
Phe	1.34	(1.24–1.45)	<0.001	1.20	(1.10–1.31)	<0.001	1.15	(1.10–1.21)	<0.001	1.13	(1.07–1.20)	<0.001
Tyr	1.65	(1.52–1.78)	<0.001	1.24	(1.13–1.36)	<0.001	1.16	(1.10–1.22)	<0.001	1.25	(1.19–1.32)	<0.001
Ala	2.15	(1.98–2.33)	<0.001	1.70	(1.55–1.86)	<0.001	1.53	(1.45–1.61)	<0.001	1.37	(1.30–1.45)	<0.001
Gly	0.57	(0.51–0.64)	<0.001	0.69	(0.61–0.78)	<0.001	0.87	(0.83–0.91)	<0.001	0.84	(0.80–0.89)	<0.001
Gln	0.83	(0.77–0.90)	<0.001	0.92	(0.83–1.01)	0.082	1.01	(0.97–1.06)	0.581	0.85	(0.80–0.89)	<0.001
Ser	0.76	(0.70–0.83)	<0.001	1.14	(1.04–1.26)	0.008	0.83	(0.79–0.87)	<0.001	0.80	(0.76–0.85)	<0.001
His	1.06	(1.01–1.13)	0.031	1.05	(0.99–1.11)	0.134	1.17	(1.09–1.25)	<0.001	0.99	(0.94–1.05)	0.739
Lys	1.35	(1.25–1.47)	<0.001	1.30	(1.19–1.43)	<0.001	1.39	(1.32–1.47)	<0.001	1.02	(0.97–1.08)	0.398
Met	1.16	(1.07–1.26)	<0.001	1.13	(1.05–1.21)	0.002	1.06	(1.00–1.11)	0.038	1.03	(0.97–1.08)	0.326
Thr	1.18	(1.10–1.28)	<0.001	1.10	(1.00–1.21)	0.042	1.02	(0.97–1.07)	0.404	1.12	(1.07–1.18)	<0.001
Asn	0.79	(0.72–0.86)	<0.001	1.05	(0.95–1.15)	0.321	0.87	(0.83–0.91)	<0.001	0.89	(0.84–0.93)	<0.001
Cit	0.84	(0.77–0.93)	<0.001	1.10	(1.00–1.20)	0.053	0.97	(0.92–1.01)	0.160	0.93	(0.88–0.99)	0.013
Arg	1.00	(0.92–1.08)	0.993	1.13	(1.03–1.24)	0.009	1.12	(1.07–1.17)	<0.001	0.98	(0.93–1.03)	0.482
Pro	1.46	(1.36–1.57)	<0.001	1.34	(1.24–1.46)	<0.001	1.31	(1.25–1.38)	<0.001	1.17	(1.11–1.24)	<0.001
Orn	1.16	(1.07–1.26)	<0.001	1.23	(1.14–1.33)	<0.001	1.16	(1.11–1.22)	<0.001	1.04	(0.98–1.09)	0.205
Index 1	3.23	(2.92–3.57)	<0.001	2.10	(1.89–2.33)	<0.001	1.69	(1.61–1.79)	<0.001	1.52	(1.44–1.61)	<0.001
Index 2	2.37	(2.16–2.61)	<0.001	1.66	(1.50–1.84)	<0.001	1.53	(1.45–1.62)	<0.001	1.48	(1.39–1.57)	<0.001

Values are odds ratios (95% confidence intervals) as a continuous variable per SD increment for developing metabolic syndrome, *DM*, dyslipidemia, or hypertension from logistic regressions

Next we confirmed the capabilities of the PFAA to evaluate their association with lifestyle-related diseases after excluding the probable cross-over effects among diseases on the single PFAA level and each PFAA index (Table 5). For this purpose, the diseases were adjusted for different relevant factors for each disease separately. After such adjustments, the typical pattern of association for BCAA persisted with all diseases except hypertension. In contrast, after adjustments, the odds ratios of AAA lost significant positive associations for all diseases except Trp in dyslipidemia. On the other hand, most of the gluconeogenesis-related amino acids retained their typical pattern of association with the diseases. Also, positive associations of both PFAA indexes remained statistically significant with all lifestyle-related diseases (Table 5).

**Table 5:** The odds ratios for evaluating metabolic syndrome, DM, dyslipidemia, or hypertension adjusting for additional factors

	^a^Metabolic syndrome			^b^DM			^c^Dyslipidemia			^d^Hypertension		
	Odds ratio		*p*-value	Odds ratio		*p*-value	Odds ratio		*p*-value	Odds ratio		*p*-value
Val	1.48	(1.33–1.64)	<0.001	1.67	(1.50–1.87)	<0.001	1.48	(1.39–1.58)	<0.001	1.01	(0.94–1.08)	0.816
Leu	1.39	(1.25–1.54)	<0.001	1.59	(1.41–1.78)	<0.001	1.57	(1.47–1.68)	<0.001	0.95	(0.88–1.02)	0.137
Ile	1.46	(1.32–1.62)	<0.001	1.52	(1.36–1.69)	<0.001	1.43	(1.34–1.53)	<0.001	1.03	(0.96–1.11)	0.414
Trp	1.07	(0.97–1.18)	0.169	0.88	(0.79–0.98)	0.024	1.25	(1.18–1.32)	<0.001	0.98	(0.92–1.04)	0.471
Phe	0.96	(0.87–1.07)	0.470	1.10	(1.00–1.21)	0.051	1.01	(0.95–1.07)	0.794	0.98	(0.92–1.04)	0.503
Tyr	1.08	(0.98–1.19)	0.115	1.07	(0.97–1.18)	0.164	0.96	(0.91–1.02)	0.151	1.01	(0.95–1.08)	0.740
Ala	1.70	(1.55–1.87)	<0.001	1.47	(1.33–1.63)	<0.001	1.34	(1.26–1.42)	<0.001	1.08	(1.01–1.15)	0.021
Gly	0.76	(0.67–0.86)	<0.001	0.76	(0.67–0.86)	<0.001	0.96	(0.91–1.01)	0.079	0.93	(0.88–0.99)	0.020
Gln	0.77	(0.70–0.84)	<0.001	0.93	(0.84–1.03)	0.161	1.01	(0.96–1.06)	0.688	0.89	(0.84–0.94)	<0.001
Ser	0.82	(0.74–0.91)	<0.001	1.27	(1.14–1.40)	<0.001	0.88	(0.84–0.93)	<0.001	0.87	(0.82–0.93)	<0.001
His	1.05	(0.98–1.13)	0.137	1.03	(0.95–1.12)	0.432	1.05	(0.99–1.11)	0.109	0.89	(0.81–0.97)	0.007
Lys	1.14	(1.04–1.26)	0.005	1.24	(1.12–1.36)	<0.001	1.25	(1.18–1.32)	<0.001	0.91	(0.86–0.97)	0.002
Met	1.04	(0.97–1.11)	0.328	1.09	(1.02–1.17)	0.017	0.98	(0.93–1.04)	0.593	0.95	(0.89–1.02)	0.127
Thr	1.17	(1.08–1.27)	<0.001	1.05	(0.95–1.15)	0.350	0.98	(0.93–1.03)	0.421	1.06	(1.00–1.12)	0.061
Asn	0.84	(0.76–0.92)	<0.001	1.08	(0.98–1.19)	0.103	0.90	(0.85–0.95)	<0.001	0.92	(0.87–0.98)	0.008
Cit	0.98	(0.88–1.08)	0.648	1.13	(1.03–1.24)	0.011	1.02	(0.97–1.08)	0.463	1.04	(0.98–1.10)	0.253
Arg	1.01	(0.92–1.11)	0.824	1.15	(1.05–1.27)	0.003	1.11	(1.05–1.16)	<0.001	0.99	(0.93–1.05)	0.658
Pro	1.38	(1.27–1.50)	<0.001	1.25	(1.15–1.36)	<0.001	1.20	(1.14–1.27)	<0.001	1.05	(0.99–1.12)	0.137
Orn	1.06	(0.97–1.17)	0.217	1.18	(1.09–1.29)	<0.001	1.11	(1.05–1.18)	<0.001	0.99	(0.93–1.05)	0.716
Index 1	1.88	(1.68–2.11)	<0.001	1.83	(1.62–2.06)	<0.001	1.36	(1.28–1.45)	<0.001	1.12	(1.04–1.20)	0.003
Index 2	1.44	(1.29–1.60)	<0.001	1.37	(1.22–1.53)	<0.001	1.24	(1.16–1.33)	<0.001	1.12	(1.04–1.21)	0.002

Values are odds ratios (95% confidence intervals) as a continuous variable per SD increment for developing metabolic syndrome, *DM,* dyslipidemia, or hypertension from logistic regressions

^a^Adjusted for age, gender and BMI

^b^Adjusted for age, gender, BMI, LDL-C, HDL-C, TG, SBP and DBP

^c^Adjusted for age, gender, BMI, FPG, HbA1c, SBP and DBP

^d^Adjusted for age, gender, BMI, FPG, HbA1c, LDL-C, HDL-C and TG

## Discussion

In this study, we have examined the relationship between PFAA profiles and lifestyle-related disease risks with more than 8000 subjects, so far the largest Asian population for such type of studies. We confirmed that PFAA profiles and the indexes generated from them are useful for evaluating lifestyle-related diseases. Our analysis showed that concentrations of several single PFAA and PFAA indexes could identify the subjects who are at risks for metabolic syndrome, DM, dyslipidemia, and hypertension.

Recently, an increased number of reports on the association between PFAA profiles and lifestyle-related diseases are being observed [11–13]. For example, BCAAs especially Val and Leu, have been proposed as a cardiometabolic risk marker independent of BMI category [11]. Furthermore, BCAAs have been linked to the metabolic syndrome, insulin resistance or type 2 DM [12, 17]. Interactions of excess BCAA and lipids may lead to the development of β-cell dysfunction, which drives the transition from the obese, insulin-resistant state to type 2 DM [17]. With respect to such factors like insulin sensitivity and visceral obesity, there are differences between Asian and Western populations [18, 19]. But the number of studies focusing on these issues is limited [12, 20].

As revealed in this study, there was a consistent increase in the concentrations of BCAA in all four lifestyle-related diseases. Also, BCAA exhibited significant positive association with all those diseases. Such an observation is consistent with the findings of other research works [12, 13]. As the underlying mechanisms, it has been suggested that a decrease in insulin activity and utilization of amino acids in muscles induces reduced uptake of BCAAs into muscles resulting in increased BCAA levels in lifestyle-related diseases [21]. Newgard et al. postulated that the rise in circulating BCAA in obese and insulin-resistant subjects is partially caused by a decline in their catabolism in adipose tissue possibly via down-regulation of the BCAA catabolic enzymes through the suppression of peroxisome proliferator-activated receptor-γ (PPAR-γ) signaling in such metabolic adaptation [17]. All these might have been reflected in our findings of significant positive association of BCAA with lifestyle-related diseases. However, although metabolic syndrome, DM, dyslipidemia, and hypertension showed positive association with BCAA in this study after adjusting for age and gender, hypertension lost the association after further adjustments for the disease-specific factors. These results might suggest that in addition to the currently known risk factors like diet or nutrition, differing metabolism for different amino acids probably contributes to increased risk of lifestyle-related diseases.

The underlying mechanisms for the observed elevation in AAA concentrations remain unclear. However, repression of tyrosine aminotransferase during states of insulin resistance and DM may result in the increased levels of circulating Tyr and Phe as observed in our study [12, 22].

In our analysis, metabolic syndrome, dyslipidemia, and hypertension showed similar patterns for association with gluconeogenesis-related amino acids. The diseases positively correlated with Ala, and negatively with Gly, Gln and Ser except Ser in DM and Gln in dyslipidemia. It was reported that under condition of hyperinsulinemia, increased protein turnover causes greater supply of gluconeogenic amino acids to the liver, leading to the reductions of Gly, Gln and Ser [23]. Additionally, gluconeogenesis precursors including Ala have been reported to rise in those with deteriorating glucose tolerance [24].

The present study has confirmed that PFAA indexes which reflect VFA and insulin resistance, respectively [12], could assess lifestyle-related diseases investigated in the largest Japanese population. In this study, index 1 was associated with most of the variables more positively than any single amino acid, and the tendency was more apparent than that of index 2. The association pattern of the indexes and PFAA with each lifestyle-related disease is distinct. Although hypertension showed no association with BCAA and AAA after adjustments for exclusion of cross-over effects among diseases, corresponding odds ratios for both index 1 and index 2 were significant. Of the diseases examined, only dyslipidemia showed higher odds ratios with BCAA than with index 1. These results revealed the distinct contribution of amino acid metabolism to the risk of each lifestyle-related disease. Further analysis of the relationship between the amino acid metabolisms and disease risk may lead to the understanding of pathophysiology, diagnosis and prevention of lifestyle-related diseases.

Our analysis showed moderate correlation between PFAA indexes and hypertension, which was previously shown to be weak [12]. The difference between the results might be due to a higher number of subjects in this study. However, it would be interesting to know the effects of salt intake on the relationship between hypertension and PFAA profiles in future studies.

The findings of our study should be interpreted in light of the limitation that we could not include the information related to the lifestyle factors such as physical activity and diet due to limited available data on these, which may have affected the PFAA profiles observed in this study.

## Conclusions

Our study confirmed the usefulness of PFAA profiles as markers for evaluating the risks of lifestyle-related diseases, including DM, metabolic syndrome, dyslipidemia, and hypertension in a large Asian population.

## Abbreviations

AAA: Aromatic amino acids
Ala: Alanine
Arg: Arginine
Asn: Asparagine
BCAA: Branched-chain amino acids
BMI: Body mass index
Cit: Citrulline
DBP: Diastolic blood pressure
DM: Diabetes mellitus
FPG: Free plasma glucose
Gln: Glutamine
Gly: Glycine
HbA1c: Haemoglobin A1c
HDL-C: High-density lipoprotein cholesterol
His: Histidine
Ile: Isoleucine
LDL-C: Low-density lipoprotein cholesterol
Leu: Leucine
Lys: Lysine
Met: Methionine
Orn: Ornithine
PFAA: Plasma free amino acid
Phe: Phenylalanine
Pro: Proline
SBP: Systolic blood pressure
Ser: Serine
T-CHO: Cholesterol
TG: Triglyceride
Thr: Threonine
Trp: Tryptophane
Tyr: Tyrosine
Val: Valine
VFA: Visceral fat area
